# Age and HbA1c in Diabetes: A Negative Association Modified by Red Cell Characteristics

**DOI:** 10.3390/jcm13237487

**Published:** 2024-12-09

**Authors:** Oyuntugs Byambasukh, Munkhuchral Nordog, Bao Suya, Narkhajid Galsanjigmed, Altangadas Dashnyam, Altaisaikhan Khasag, Odgerel Tsogbadrakh, Otgonbat Altangerel

**Affiliations:** 1 Department of Endocrinology, School of Medicine, Mongolian National University of Medical Sciences, Ulaanbaatar 14210, Mongolia; narkhajid@mnums.edu.mn (N.G.); altaisaikhan@mnums.edu.mn (A.K.); 2 Department of Internal Medicine, Mongolia-Japan Hospital, Mongolian National University of Medical Sciences, Ulaanbaatar 14210, Mongolia; munkhuchral@mnums.edu.mn (M.N.); suya6535@gmail.com (B.S.); odgerel.ts@mnums.edu.mn (O.T.); 3 Department of Hematology, School of Medicine, Mongolian National University of Medical Sciences, Ulaanbaatar 14210, Mongolia; 4 Xing’an Vocational and Technical College in Inner Mongolia Ulanhot, Ulanhot 137400, China; 5 Department of Histology, School of Biomedicine, Mongolian National University of Medical Sciences, Ulaanbaatar 14210, Mongolia; altangadas@mnums.edu.mn

**Keywords:** diabetes mellitus, red cell distribution width, glycated hemoglobin A

## Abstract

**Background**: While a positive correlation between age and HbA1c has been suggested in non-diabetic individuals, warranting higher HbA1c reference ranges for older adults, evidence among individuals with diabetes is less clear and may reveal an inverse trend. This study aimed to examine the relationship between age and HbA1c in a diabetic population, considering red cell parameters and other confounding factors; **Methods**: This cross-sectional study included 268 diabetic participants from Mongolia-Japan University Hospital (mean age 57.0 ± 9.9 years, 38.8% male, median diabetes duration 8.0 years, mean HbA1c 9.2 ± 3.3%). We analyzed the association between age and HbA1c using linear regression models, adjusting for diabetic characteristics, chronic complications, inflammation markers, and red cell indices. Subgroup analyses were conducted based on red cell distribution width (RDW) median splits; **Results**: A significant negative association between age and HbA1c was observed, with an unstandardized B coefficient (95% CI) of −0.112 (−0.166; −0.058, *p* < 0.001). This association persisted after adjustment for diabetic characteristics, complications, inflammation markers, and red cell indices (−0.115, −0.179; −0.051, *p* = 0.001). Subgroup analyses indicated a stronger negative association in participants with lower RDW levels (−0.174, −0.269; −0.079, *p* < 0.001) compared to those with higher RDW (−0.080, −0.147; −0.014, *p* = 0.019), suggesting that red cell characteristics may modify this relationship. No significant interactions were identified except for RDW; **Conclusions**: Our findings reveal a distinct negative association between age and HbA1c in diabetic individuals, independent of diabetic characteristics, complications, and inflammation markers. This association is particularly pronounced in individuals with lower RDW levels, highlighting the potential role of red cell morphology in influencing HbA1c levels with aging in diabetes.

## 1. Introduction

Diabetes mellitus (DM), a chronic metabolic disorder marked by persistent hyperglycemia, represents a substantial global health burden, with escalating prevalence and related complications worldwide [1]. Hemoglobin A1c (HbA1c) serves as a critical biomarker in evaluating long-term glycemic control, guiding treatment decisions, and reducing the risk of diabetes-associated complications [2,3].

While HbA1c levels are known to change with age, the nature of these changes varies between diabetic and non-diabetic populations. In non-diabetic individuals, studies have suggested a positive correlation between age and HbA1c, potentially due to age-related alterations in red blood cell (RBC) turnover and lifespan [4,5,6,7]. These findings have prompted the development of higher age-specific HbA1c reference ranges to avoid overdiagnosis of diabetes in older adults [7]. However, among individuals with diabetes, emerging evidence indicates an inverse relationship between age and HbA1c [8,9]. This intriguing trend may reflect improved glycemic control with age or changes in diabetes management, but the underlying mechanisms remain unclear. Furthermore, the association between age and HbA1c in diabetic patients holds significant clinical implications. Understanding this relationship is crucial for tailoring treatment strategies, as older adults with diabetes may require individualized HbA1c targets to optimize management and prevent complications.

A critical factor influencing HbA1c levels is RBC morphology and function. Red cell distribution width (RDW), a measure of variability in RBC size, has been identified as a marker of systemic inflammation, oxidative stress, and abnormalities in iron metabolism [4,5]. Elevated RDW levels have been associated with worse glycemic control and cardiovascular outcomes, highlighting the potential role of RBC parameters in modifying the relationship between age and HbA1c [10]. Despite this, the interplay between RBC indices, age, and HbA1c levels in diabetes remains underexplored.

This study aims to examine the association between age and HbA1c among individuals with diabetes and to determine whether this relationship is independent of RBC parameters and other potential confounding factors. Specifically, we assessed red cell indices (RDW, MCV, RBC count, and hemoglobin), markers of iron metabolism (soluble transferrin receptor /sTfR/ and reticulocyte hemoglobin equivalent /RET-He/), chronic diabetic complications, inflammation markers (high-sensitivity C-reactive protein /Hs-CRP/, homocysteine, and ferritin), and lifestyle factors (physical activity, smoking, and alcohol use). Beyond these physiological and lifestyle factors, pharmacological interventions can also influence HbA1c levels. For instance, liraglutide, a glucagon-like peptide-1 receptor agonist, has demonstrated benefits in improving glycemic control, body composition, and lipid profiles in patients with type 2 diabetes [11]. We have adjusted our study to account for more factors influencing HbA1c, such as RBC morphology and lifestyle habits, to better understand the interplay between age and HbA1c in diabetic populations.

## 2. Materials and Methods

### 2.1. Data Source and Study Population

This cross-sectional study enrolled patients from the Mongolia-Japan University Hospital of the Mongolian National University of Medical Sciences. Participants were recruited among patients attending the Endocrinology unit between February and August of 2022, totaling 418 individuals within this 6-month period.

The inclusion criteria specified a confirmed diagnosis of diabetes, whereas exclusion criteria encompassed malignancies (*n* = 6), hematological disorders such as anemia or hemoglobinopathies (*n* = 8), renal or hepatic failure (*n* = 38), acute infections or chronic inflammatory conditions such as rheumatoid arthritis (*n* = 20), recent cardiovascular events such as myocardial infarction or stroke within the past 3 months (*n* = 3), and other types of diabetes, including type 1 diabetes mellitus (*n* = 22). After applying these criteria, 321 patients were invited to participate in the study, resulting in a final cohort of 268 individuals.

### 2.2. Data Collection

Patients participated in various aspects of the study, which included questionnaire administration, anthropometric measurements, screening for diabetic chronic complications, and the collection of laboratory blood samples.

The questionnaire involved the collection of crucial patient information, encompassing general demographics, lifestyle characteristics (such as smoking and alcohol use, frequency of leisure time physical activity a week), and medical history related to diabetes. This comprehensive history included details such as the duration of diabetes, treatment methods, treatment adherence, and self-management practices. Screening for chronic diabetic complications comprised professional fundoscopy to detect eye-related issues, diabetic foot examinations conducted by an endocrinologist, and assessments of diabetic kidney complications through measurements of microalbuminuria and serum creatinine levels. Additional inquiries concerning cardiovascular and other complications were integrated into the questionnaire and administered by the data collector.

Diabetes characteristics in this study included the type of treatment, duration of diabetes, and the presence of chronic complications such as nephropathy, retinopathy, and neuropathy. Glycemic control was categorized as either poor or good, based on whether glycated hemoglobin (HbA1c) levels were above or below 7.5% [12,13]. Anthropometric measurements included body weight, height, waist circumference, and blood pressure (in mmHg). Body mass index (BMI) was calculated by dividing weight (in kilograms) by the square of height (in square meters). Hypertension was defined based on self-reported diagnoses, the use of antihypertensive medications, and blood pressure measurements exceeding 130/80 mmHg for systolic and diastolic blood pressure, respectively [13].

### 2.3. Laboratory Measurements

XN2000 (Sysmex, Kobe, Hyogo, Japan) hematology autoanalyzer was used for complete blood count and red blood cell count; additional parameters, such as mean corpuscular volume (MCV), mean corpuscular hemoglobin (MCH), mean corpuscular hemoglobin concentration (MCHC), and red cell distribution width (RDW) were determined. This study used an immunoassay method to assess Hemoglobin A1c (HbA1c). This sophisticated technique was employed to quantify the percentage of glycated hemoglobin, providing precise measurements for a comprehensive understanding of glycemic control.

### 2.4. Statistical Analysis

The general characteristics of the study population were summarized using means with a standard deviation (SD). In cases where continuous variables did not follow a normal distribution, we presented them as mean with minimum and maximum values. Categorical variables were expressed as percentages with corresponding numbers. A Shapiro–Wilk test was used to assess the normality of continuous variables, including HbA1c, RBC parameters, and inflammatory markers. We employed several statistical tests to assess differences among variables, including a Student’s *t* test, Mann-U-Whitney test, one-way ANOVA, and Chi-square test.

We utilized Pearson’s correlation test to examine associations between variables in our study. For assessing interactions, we conducted a two-way ANOVA, investigating the relationship between age and HbA1c using subgroups. The subgroups were defined based on diabetes duration (<10 and ≥10 years), diabetic complications (with and without complications), and median-split groups of RDW and RBC. To ensure comparable group sizes and address statistical power considerations, we chose not to rely on pre-defined age and HbA1c categories. Instead, we categorized these variables based on tertiles derived from their distribution in the study population. Additionally, RBC was divided into tertiles. Moreover, we performed linear regression using both basic and adjusted models. Model 1 comprised the basic model plus diabetic characteristics (type of treatment, diabetic chronic complications, diabetes duration, and diabetes duration square) and inflammation markers (IL-6, Hs-CRP, and Homocysteine). In Model 2, we added lifestyle factors (smoking, alcohol use, and physical activity). Model 3 expanded on Model 2 by incorporating iron-metabolism-related factors (sTFR, RET-HE, and Ferritin). Finally, Model 4 extended Model 3, adding RDW-SD as an additional variable. Linear regression models were employed to evaluate the association between age and HbA1c due to their robustness in identifying relationships between continuous variables while controlling for potential confounders. This approach was chosen over alternative methods to provide a clear assessment of the independent effects of age and other factors on HbA1c levels.

For all statistical analyses, we used IBM SPSS V.28.0 (IBM, Chicago, IL, USA), and a statistical significance level was set at *p* < 0.05 for all the tests.

## 3. Results

This study included 268 participants, with a mean age of 57.0 ± 9.9 years. Of the participants, 38.8% were male (*n* = 104), and 16.4% had a lower level of education. The duration of diabetes ranged widely, with a median of 8.0 years (range 0 to 39 years). The mean HbA1c level was 9.2 ± 3.3%. Regarding treatment, 6.3% of participants did not use oral glycemic drugs (OGDs) or insulin, 66.8% used OGDs alone, 14.9% used a combination of OGDs and insulin, and 11.9% received multi-dose insulin therapy. Nearly half of the participants (47.9%) had one or more chronic complications related to diabetes, with 23.5% experiencing at least one complication and the remainder having multiple complications. The most common complications included diabetic retinopathy (26.5%), nephropathy (23.1%), neuropathy (11.9%), diabetic foot issues (13.4%), and cardiovascular complications (16.0%). Participants had a mean BMI of 29.7 kg/m^2^, with 39.9% classified as obese (BMI > 30.0) and 40.7% as overweight (BMI 25–29). The mean systolic blood pressure (SBP) was 130.9 ± 16.7 mmHg, and 58.6% of participants had hypertension. Additionally, 22.4% reported smoking, and 22.0% reported alcohol use.

Age-related differences in study characteristics are detailed in Table 1. Although gender distribution varied across age tertiles, these differences did not reach statistical significance (*p* = 0.059). Significant differences were observed in diabetes duration among the tertiles (*p* < 0.001), with participants in T3 having a significantly longer median diabetes duration compared to those in T1 and T2. HbA1c levels also differed significantly across tertiles (*p* = 0.003), with T1 showing the highest mean levels and T3 the lowest. While there were differences in insulin use among the age groups, these were not statistically significant. Complication rates varied across age tertiles but did not reach statistical significance. BMI values differed slightly across age groups, though these differences were not statistically significant (*p* = 0.765). SBP levels also varied across tertiles but were not statistically significant (*p* = 0.054). In terms of smoking and alcohol use, there was a notable trend where older participants reported healthier behaviors. Smoking prevalence was highest in T1 and decreased across tertiles (*p* = 0.003). Similarly, alcohol use was more common in T1 and T2 compared to T3 (*p* = 0.002).

Table 2 shows laboratory measurements, focusing on red cell parameters across age tertiles. Red blood cell (RBC) counts were significantly different across age tertiles (*p* < 0.001), with T1 showing the highest counts, followed by T2 and T3. Conversely, mean corpuscular volume (MCV) and red cell distribution width (RDW) increased with age, and these differences were statistically significant. While hemoglobin (HGB) levels tended to decrease with age and mean corpuscular hemoglobin (MCH) increased, these differences were not statistically significant. Other parameters, including hematocrit (HCT) and RET-HE, showed no significant age-related differences. Furthermore, markers of iron metabolism, such as ferritin and soluble transferrin receptor (sTfR), along with inflammatory markers like high-sensitivity C-reactive protein (Hs-CRP) and IL-6, did not show significant differences across age tertiles (Table 2).

Correlations between HbA1c and red cell parameters indicated statistically significant inverse relationships between HbA1c and RDW-SD (r = −0.257, *p* < 0.001) as well as RDW-CV (r = −0.159, *p* = 0.001). No significant correlations were observed between HbA1c and other red cell parameters, including RBC, HGB, HCT, MCV, and MCH. Notably, some strong correlations existed among red cell parameters (e.g., RBC and HGB), but these did not correlate strongly with HbA1c, suggesting limited multicollinearity between RDW-SD, RDW-CV, and other red cell indices. A weak yet significant positive correlation was observed between HbA1c and the number of diabetic complications (r = 0.128, *p* = 0.038) and diabetes duration (r = 0.213, *p* < 0.001).

The primary objective was to assess whether the association between age and HbA1c was independent of red cell parameters and other factors. Interaction effects were tested for diabetes duration, complications, smoking status, and median splits of RDW and RBC. An interaction effect from RDW (*p* = 0.041) was observed in the age–HbA1c association, prompting separate analyses for lower and higher RDW groups. A significant negative association was observed between age and HbA1c in the unadjusted model (Table 3). This association strengthened after adjusting for diabetic characteristics (Model 1), while further adjustment for RDW-SD slightly weakened it (Model 2). The inclusion of iron-metabolism-related factors had minimal impact (Model 3), while the addition of inflammation markers slightly strengthened the association (Model 4). Adjusting for lifestyle characteristics somewhat reduced the association (Model 5). These results suggest that, in diabetic patients, the association between age and HbA1c is predominantly negative, though it may be influenced by diabetic characteristics and inflammation markers, with lifestyle modifications partially counteracting this effect.

**Figure 1:**
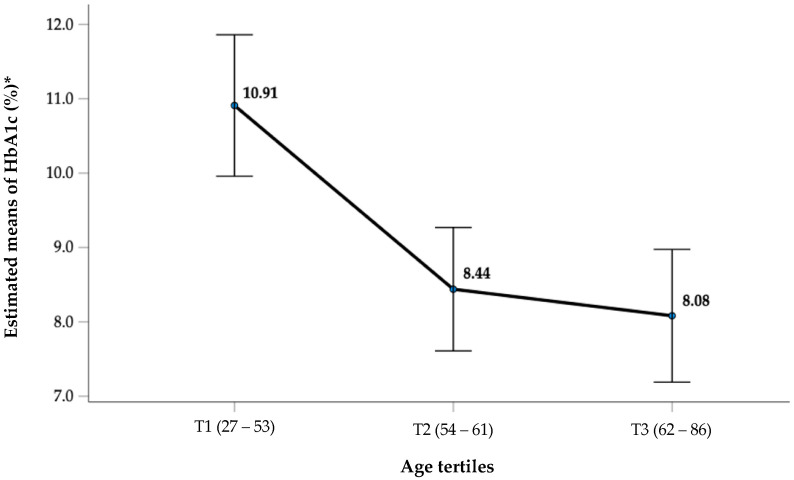
Estimated and adjusted means of HbA1c across age tertiles. Error bars represent 95% confidence intervals. Statistical significance was observed for comparisons between T1 and T2 (*p* = 0.002) and between T2 and T3 (*p* = 0.035), highlighting a significant decline in HbA1c with advancing age. Note: * adjusted for diabetes duration, diabetic chronic complications, inflammation markers, iron metabolism-related factors, lifestyle characteristics, and RDW-SD.

As shown in Figure 1, there is a clear, progressive decline in HbA1c levels from the youngest age tertile (T1: 27–53 years) to the oldest tertile (T3: 62–86 years), even after adjusting for confounders. The highest estimated mean HbA1c (10.91%) was observed in the youngest tertile, while the lowest (8.08%) was in the oldest tertile. This suggests that younger individuals in this diabetic population tend to have poorer glycemic control than older individuals. This finding may reflect differences in diabetes management, lifestyle factors, or disease adaptation with age. The overall trend supports the hypothesis that age is inversely associated with HbA1c levels in this cohort.

**Figure 2:**
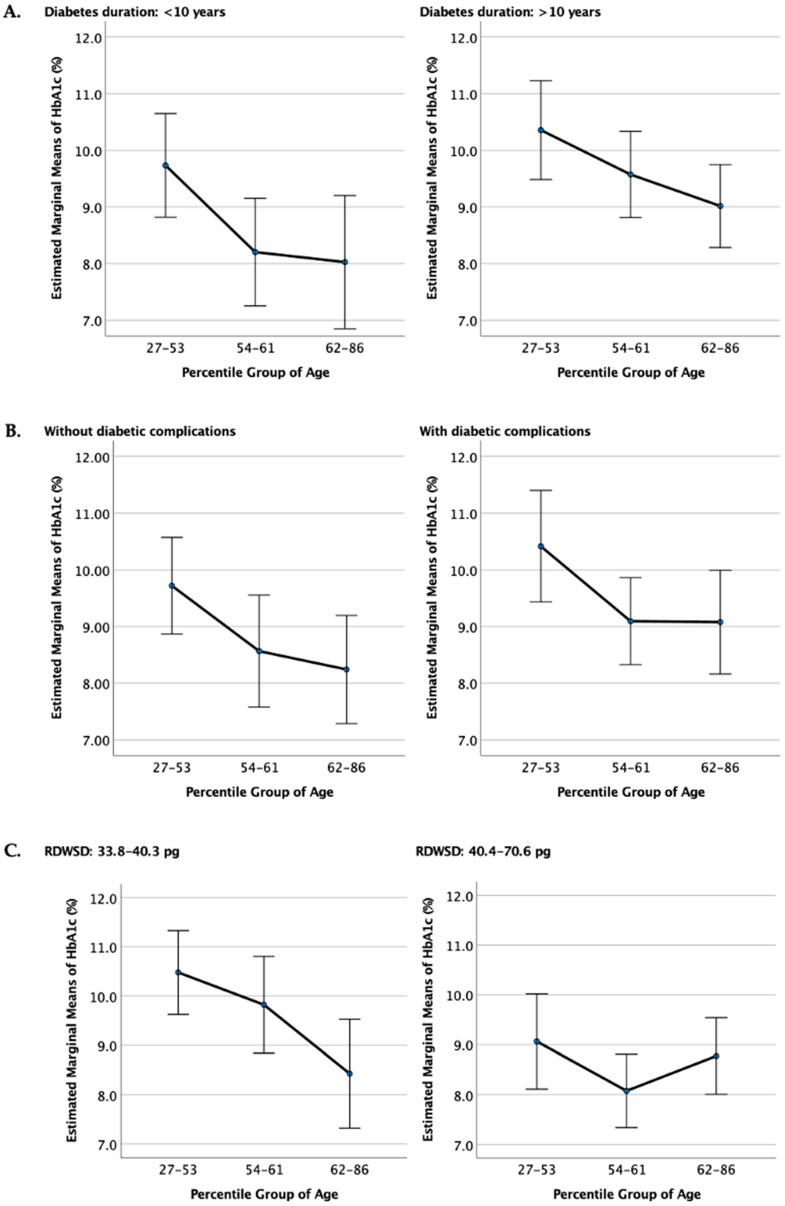
Stratification by diabetes duration, complications, and RDW-SD groups. (**A**) Estimated marginal means of HbA1c (%) stratified by diabetes duration (<10 years and >10 years). Error bars represent 95% confidence intervals. Significant differences between age tertiles were observed in both groups (*p* < 0.05). (**B**) Estimated marginal means of HbA1c (%) stratified by the presence of diabetic complications. Error bars represent 95% confidence intervals. Significant differences between age tertiles were observed in both subgroups (*p* < 0.05). (**C**) Estimated marginal means of HbA1c (%) stratified by RDW-SD levels. Trends in HbA1c across age tertiles were observed, but statistical significance was not reached for comparisons within RDW-SD groups.

Figure 2 explores how diabetes duration, presence of complications, and RDW-SD levels impact the age–HbA1c relationship. These subgroup analyses are crucial as they help identify whether specific patient characteristics modify the association between age and HbA1c, providing a clearer understanding of the factors influencing glycemic control across different age groups.

Participants were divided based on diabetes duration to assess if HbA1c trends with age differed between those with shorter vs. longer diabetes histories. This analysis is essential as diabetes duration can impact disease progression, treatment adherence, and physiological adaptations. In both groups (<10 years and >10 years), HbA1c levels declined with age. However, individuals with a shorter diabetes duration (<10 years) had higher HbA1c levels across all age groups compared to those with longer diabetes duration (>10 years). This suggests that, despite age-related improvements, younger individuals with recent diabetes diagnoses may require more intensive management to achieve optimal glycemic control. The results imply that disease duration moderates the age–HbA1c relationship, with longer duration potentially reflecting better diabetes adaptation or treatment stability. We stratified participants based on the presence or absence of diabetic complications to understand if complications influence the age–HbA1c association. This is relevant as complications often necessitate stricter glycemic control measures, potentially affecting HbA1c levels. Both groups (with and without complications) showed a decline in HbA1c with age. However, those without complications displayed consistently higher HbA1c levels across age groups compared to those with complications. This might indicate that individuals with complications are more likely to engage in stricter glycemic control or receive more intensive medical oversight, leading to lower HbA1c values. The findings suggest that while age remains inversely associated with HbA1c, the presence of complications could motivate enhanced management, thus impacting HbA1c outcomes. RDW-SD levels were analyzed to determine whether red cell distribution width affects the age–HbA1c association. RDW-SD reflects red cell variation and can indicate underlying inflammation or iron metabolism abnormalities, which are known to impact HbA1c levels. In both low- and high-RDW-SD groups, HbA1c levels decreased with age. However, the decline was more pronounced in the low-RDW-SD group. This suggests that higher RDW-SD (reflecting increased red cell variability) may attenuate the age-related decline in HbA1c. This could be due to red cell turnover or inflammatory processes that influence HbA1c independently of age. These results highlight the potential role of hematological factors, like RDW-SD, in moderating the age–HbA1c relationship and underscore the importance of considering red cell parameters in HbA1c interpretation.

**Figure 3:**
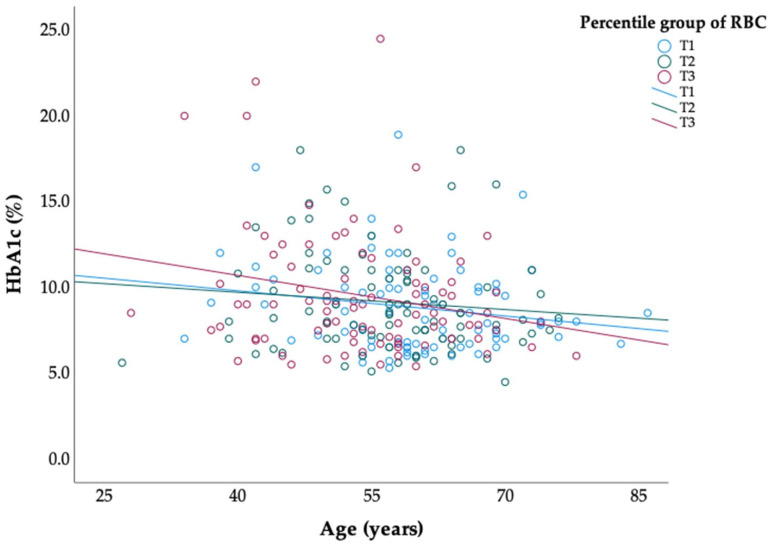
Association of age and HbA1c by RBC percentile groups. Scatterplot illustrating the association between age and HbA1c (%) across RBC percentile groups (T1, T2, T3). Trend lines indicate a significant negative association between age and HbA1c (*p* < 0.05), while differences between RBC groups and their interaction with age were not statistically significant (*p* = 0.637 and *p* = 0.942, respectively).

Figure 3 presents the association between age and HbA1c by stratifying participants into RBC percentile groups. Given that RBC counts affect HbA1c through their impact on glycation rates, analyzing this relationship by RBC levels allows us to evaluate if HbA1c trends with age are consistent across different red cell profiles. Across all RBC tertiles, there was a negative association between age and HbA1c, although the slope of decline varied slightly between groups. A significant negative association between age and HbA1c was observed (β = −0.112; 95% CI: −0.166, −0.058; *p* < 0.001). Subgroup analyses revealed consistent negative trends across RBC percentile groups, although statistical differences between these groups were not significant (*p* = 0.637). Furthermore, the interaction between age and RBC groups did not reach statistical significance (*p* = 0.942). Individuals with higher RBC counts (T3) tended to have slightly higher HbA1c levels than those in the lower RBC groups, but the trend of decreasing HbA1c with age was consistent. This suggests that, while RBC levels contribute to HbA1c variability, they do not significantly alter the age-related decline in HbA1c. This finding supports the hypothesis that the inverse association between age and HbA1c is largely independent of RBC count variations, reinforcing age as a key factor in glycemic control trends among diabetic individuals.

## 4. Discussion

This study demonstrated a significant negative association between age and HbA1c levels among individuals with diabetes, contrasting with findings in non-diabetic populations where HbA1c levels typically exhibit a positive association with age. This negative relationship persisted even after adjusting for various confounding factors, including diabetes duration, presence of chronic diabetic complications, and RBC indices. Interestingly, further adjustment for lifestyle factors, such as smoking and physical activity, attenuated this association, suggesting that lifestyle modifications may play a moderating role in glycemic control among older adults with diabetes.

These findings underscore the complex interplay between aging, diabetes, and hematological changes. In non-diabetic populations, HbA1c levels generally increase with age due to age-associated changes in RBC turnover and lifespan [14,15,16]. However, our results suggest that diabetes modifies this trajectory, likely through mechanisms related to both disease pathology and altered RBC characteristics. Specifically, older participants in our study exhibited lower HbA1c levels, which may indicate improved glycemic control with advancing age or reflect age-related changes in RBC morphology and longevity in the diabetic state. This trend is consistent with other studies showing that older adults with diabetes tend to have lower HbA1c levels, potentially due to a combination of better diabetes management, lifestyle adjustments, and alterations in hematological parameters [9,17,18].

Our findings contribute to the ongoing discourse on HbA1c variability and its determinants in diabetic individuals. Previous studies on age-related changes in RBC morphology and function in diabetes have documented that chronic hyperglycemia induces alterations in RBC structure, including increased RBC distribution width (RDW), mean corpuscular volume (MCV), and the formation of deformed cells such as echinocytes and spherocytes [19,20]. These morphological changes are thought to be driven by oxidative stress and lipid peroxidation in diabetes, leading to compromised RBC integrity and altered HbA1c readings [15,21]. In our study, we observed a negative association between HbA1c and both MCV and RDW, indicating that red cell morphological changes play a significant role in HbA1c variability among diabetic patients. These findings align with prior research suggesting that increased RBC turnover and morphological deformations in diabetes can lead to lower HbA1c levels, particularly in older individuals [5,10,19,22].

Furthermore, our analysis showed that diabetes duration and the presence of complications strengthened the negative association between age and HbA1c. This finding is particularly relevant, as it implies that prolonged exposure to hyperglycemia and the consequent development of diabetic complications may drive physiological adaptations in RBC parameters, impacting HbA1c measurements. Studies have reported that long-term hyperglycemia results in a progressive decline in biconcave disk-shaped RBCs and an increase in deformed RBCs, such as acanthocytes, echinocytes, and spherocytes [20,21]. These deformations can affect the glycation process, as deformed RBCs may have a shorter lifespan, reducing their exposure time to glucose and subsequently lowering HbA1c levels [15,16]. Thus, in diabetic populations, the cumulative effect of disease duration and complications on RBC morphology could partially explain the observed decline in HbA1c with advancing age.

Our findings also support previous observations on the complex association between HbA1c and RDW in diabetic versus non-diabetic individuals. For instance, studies have found positive correlations between HbA1c and RDW in non-diabetic populations, whereas an inverse association is often reported in diabetic populations [10,19,23,24,25]. In our study, we observed a negative relationship between HbA1c and RDW in diabetic patients, consistent with the hypothesis that diabetes-related RBC alterations mediate this relationship. High RDW, often indicative of inflammation or oxidative stress, could lead to enhanced RBC turnover, resulting in lower HbA1c levels [10,23]. This is supported by research suggesting that inflammatory markers, including high-sensitivity C-reactive protein (Hs-CRP) and homocysteine, correlate with elevated RDW in diabetic patients, potentially impacting HbA1c levels through accelerated RBC degradation and altered glycation rates [6,8,19]. Although the difference in HbA1c levels between tertiles 2 and 3 was minimal, it remains statistically significant. Even small differences in HbA1c are clinically meaningful, as cumulative reductions over time can help lower the risk of diabetes-related complications, especially in high-risk populations.

Notably, our study emphasizes the importance of lifestyle factors in the age–HbA1c relationship. Adjustments for non-smoking status, increased physical activity, and lower alcohol consumption attenuated the negative association between age and HbA1c, underscoring the potential benefits of lifestyle modifications on glycemic control. This finding is supported by literature suggesting that lifestyle interventions can improve glycemic outcomes by reducing inflammation and enhancing RBC integrity, thereby stabilizing HbA1c levels in older diabetic individuals [3,17,26]. Given the potential for HbA1c variability in diabetes, age-specific reference values might be beneficial to avoid overtreatment and associated risks, particularly in elderly populations.

This study has implications for the interpretation of HbA1c in diabetic populations, particularly among older adults. The current HbA1c reference ranges do not account for age-related hematological changes in diabetes, which could lead to potential misinterpretation of glycemic control in elderly patients. This is consistent with studies advocating for age-specific HbA1c cutoffs to prevent unnecessary treatment intensification and its associated complications in older adults [7,9,27]. Given the negative association observed in our study, revisiting HbA1c targets in older diabetic individuals may improve individualized diabetes care and prevent adverse outcomes from overtreatment. Furthermore, a limitation of our study is that the potential impact of menopause on HbA1c variability was not directly assessed. Hormonal changes during perimenopause and menopause are known to influence glucose metabolism, and these changes may have contributed to differences in HbA1c trends observed in the first age tertile. Future studies should consider including menopausal status to provide a more comprehensive understanding of sex-specific factors affecting glycemic control in diabetic populations.

Overall, this study contributes to a growing body of evidence highlighting the need to consider age, RBC indices, and lifestyle factors in assessing HbA1c levels among diabetic patients. Future research is warranted to explore the mechanisms underlying these associations further and to evaluate the clinical utility of incorporating age-adjusted HbA1c reference ranges in diabetes management. By accounting for age and related red cell variations, clinicians may enhance the accuracy of HbA1c interpretation and optimize therapeutic interventions for older diabetic patients.

## 5. Conclusions

Our study challenges the prevailing assumption of a positive age–HbA1c association by revealing a negative association between age and HbA1c in individuals with diabetes. These findings suggest that diabetes-related alterations in red cell morphology and lifespan may modify this relationship, highlighting the potential need for age-specific HbA1c reference values in diabetic populations. Further investigation is necessary to better understand these dynamics and refine diabetes management strategies for older adults.

## Figures and Tables

**Table 1:** General characteristics of the study population.

Findings	Total Population	Age Groups (Tertiles, T1–3)			
		T1	T2	T3	*p* Value
Age (years)	57.0 ± 9.9	46.0 ± 5.7	57.6 ± 2.3	67.9 ± 5.0	-
Gender: male, % (*n*)	38.8 (104)	49.4 (44)	36.5 (35)	30.1 (25)	0.059
Diabetes duration (years)	8.0 (0–39)	5.9 (0–23)	7.7 (0–27)	10.8 (0–39)	**<0.001**
HbA1c (%)	9.2 ± 3.3	10.2 ± 4.0	8.9 ± 3.1	8.6 ± 2.4	**0.003**
DM treatment: insulin use, % (*n*)	26.8 (72)	23.6 (21)	30.2 (29)	26.5 (22)	0.188
DM chronic complications, % (*n*)					
Diabetic retinopathy	26.5 (71)	20.2 (18)	32.3 (31)	26.5 (22)	0.178
Diabetic nephropathy	23.1 (62)	15.7 (14)	27.1 (26)	26.5 (22)	0.128
Diabetic neuropathy	11.9 (32)	10.1 (9)	18.8 (18)	6.0 (5)	**0.026**
Diabetic foot	13.4 (36)	12.4 (11)	13.5 (13)	14.5 (12)	0.921
Cardiovascular complications	16.0 (43)	10.1 (9)	24.0 (23)	13.3 (11)	**0.026**
BMI (kg/m^2^)	29.7 ± 5.0	29.9 ± 5.0	29.4 ± 5.0	30.0 ± 5.1	0.765
SBP (mmHg)	130.9 ± 16.7	127.1 ± 16.0	130.7 ± 17.0	135.0 ± 16.4	0.054
Smoking use, % (*n*)	22.4 (60)	32.6 (29)	22.9 (22)	10.8 (9)	**0.003**
Alcohol use, % (*n*)	22.0 (59)	28.1 (25)	28.1 (27)	8.4 (7)	**0.002**

Data are presented as mean ± SD or mean (minimum to maximum) and number (percentages, %). Bold values denote statistical significance at the *p* < 0.05 level. Note: SD, standard deviation.

**Table 2:** Laboratory measurements of the study population.

Findings	Total Population	Age Groups (Tertiles, T1–3)			
		T1	T2	T3	*p* Value
RBC (×10^12^/L)	4.96 ± 0.58	5.18 ± 0.65	4.90 ± 0.51	4.80 ± 0.52	**<0.001**
HGB (g/dL)	14.5 ± 1.7	14.7 ± 2.0	14.4 ± 1.6	14.3 ± 1.6	0.234
HCT (%)	41.9 ± 4.3	42.6 ± 4.5	41.8 ± 4.1	41.3 ± 4.2	0.129
MCV (fL)	84.8 ± 4.9	83.4 ± 5.7	85.3 ± 4.5	85.9 ± 4.2	**0.002**
MCH (pg)	29.4 ± 2.1	29.0 ± 2.6	29.4 ± 1.8	29.7 ± 1.8	0.083
RDW-SD (pg)	40.5 ± 3.9	39.4 ± 2.7	41.1 ± 4.3	41.1 ± 4.2	**0.002**
RDW-CV (%)	13.1 ± 1.3	13.0 ± 1.3	13.2 ± 1.6	13.1 ± 0.9	0.480
RET-HE (pg)	28.3 ± 2.5	27.8 ± 3.5	28.2 ± 2.2	28.7 ± 1.6	0.252
Hs-CRP (mg/L)	0.78 (0.10–11.20)	0.30 (0.10–4.50)	1.10 (0.10–11.20)	0.88 (0.10–11.20)	0.136
Ferritin (ng/mL)	273.5 (17.0–1005.0)	293.2 (20.0–1005.0)	298.8 (17.0–1005.0)	229.5 (20.0–1005.0)	0.514
Homocysteine (µmol/L)	10.19 ± 3.49	10.94 ± 3.49	10.26 ± 3.21	9.48 ± 3.68	0.179
IL-6 (pg/mL)	6.48 (0.10–41.0)	7.68 (0.30–41.0)	6.84 (0.40–41.0)	5.01 (0.10–41.0)	0.484
sTfR (µg/mL)	14.43 ± 5.55	13.10 ± 3.15	15.45 ± 7.12	14.55 ± 5.26	0.166

Data are presented as mean ± SD or mean (minimum to maximum) and number (percentages, %). Bold values denote statistical significance at the *p* < 0.05 level. Note: SD, standard deviation.

**Table 3:** Association between age and HbA1c.

Models	Unstandardized Beta Coefficient, HbA1c			
	Beta Coefficient	95% CI		*p* Value
		Lower Bound	Upper Bound	
**Total population**				
Basic (unadjusted)	−0.112	−0.166	−0.058	**<0.001**
Model 1. Diabetic characteristics and Inflammation markers	−0.129	−0.187	−0.071	**<0.001**
Model 2. Lifestyle characteristics	−0.125	−0.184	−0.065	**<0.001**
Model 3. Iron metabolism-related factors	−0.123	−0.184	−0.062	**<0.001**
Model 4. Red cell indices	−0.115	−0.179	−0.051	**0.001**
**Lower median group of RDW**				
Basic (unadjusted)	−0.174	−0.269	−0.079	**0.001**
Model 1. Diabetic characteristics and Inflammation markers	−0.180	−0.285	−0.075	**0.001**
Model 2. Lifestyle characteristics	−0.178	−0.287	−0.069	**0.002**
Model 3. Iron metabolism-related factors	−0.172	−0.286	−0.059	**0.004**
Model 4. Red cell indices	−0.160	−0.282	−0.037	**0.012**
**Higher median group of RDW**				
Basic (unadjusted)	−0.080	−0.147	−0.014	**0.019**
Model 1. Diabetic characteristics and Inflammation markers	−0.113	−0.190	−0.035	**0.005**
Model 2. Lifestyle characteristics	−0.109	−0.189	−0.029	**0.009**
Model 3. Iron metabolism-related factors	−0.108	−0.191	−0.025	**0.012**
Model 4. Red cell indices	−0.101	−0.188	−0.014	**0.023**

Linear regression analysis. Data are expressed as an unstandardized beta coefficient with a 95% confidence interval (95% CI). Model 1: Basic model + diabetic characteristics (type of treatment, diabetic chronic complications, diabetes duration, and diabetes duration square) and Inflammation markers (IL-6, Hs-CRP, and Homocysteine). Model 2: Model 1 + Lifestyle characteristics (smoking, alcohol use and physical activity). Model 3: Model 2 + Iron metabolism-related factors (sTFR, RET-HE, and Ferritin). Model 4: Model 3 + Red cell indices (RDW).

## Data Availability

The data used to support the findings of this study are available from the corresponding author upon request.
